# Lenvatinib prevents liver fibrosis by inhibiting hepatic stellate cell activation and sinusoidal capillarization in experimental liver fibrosis

**DOI:** 10.1111/jcmm.16363

**Published:** 2021-02-20

**Authors:** Hiroyuki Ogawa, Kosuke Kaji, Norihisa Nishimura, Hirotetsu Takagi, Koji Ishida, Hiroaki Takaya, Hideto Kawaratani, Kei Moriya, Tadashi Namisaki, Takemi Akahane, Hitoshi Yoshiji

**Affiliations:** ^1^ Department of Gastroenterology Nara Medical University Nara Japan

**Keywords:** angiogenesis, hepatic stellate cell, lenvatinib, liver fibrosis, PDGF, VEGF

## Abstract

Molecular targeted agents are pharmacologically used to treat liver fibrosis and have gained increased attention. The present study examined the preventive effect of lenvatinib on experimental liver fibrosis and sinusoidal capillarization as well as the in vitro phenotypes of hepatic stellate cells. LX‐2, a human stellate cell line, was used for in vitro studies. In vivo liver fibrosis was induced in F344 rats using carbon tetrachloride by intraperitoneal injection for 8 weeks, and oral administration of lenvatinib was started two weeks after initial injection of carbon tetrachloride. Lenvatinib restrained proliferation and promoted apoptosis of LX‐2 with suppressed phosphorylation of extracellular signal‐regulated kinase 1/2 and AKT. It also down‐regulated *COL1A1*, *ACTA2* and *TGFB1* expressions by inhibiting the transforming growth factor‐β1/Smad2/3 pathway. Treatment with lenvatinib also suppressed platelet‐derived growth factor‐BB‐stimulated proliferation, chemotaxis and vascular endothelial growth factor‐A production, as well as basic fibroblast growth factor‐induced LX‐2 proliferation. In vivo study showed that lenvatinib attenuated liver fibrosis development with reduction in activated hepatic stellate cells and mRNA expression of profibrogenic markers. Intrahepatic neovascularization was ameliorated with reduced hepatic expressions of *Vegf1*, *Vegf2* and *Vegfa* in lenvatinib‐treated rats. Collectively, these results suggest the potential use of lenvatinib as a novel therapeutic strategy for liver fibrosis.

## 1 INTRODUCTION

Liver fibrosis is a chronic pathological abnormality characterized by collapsed hepatic parenchyma and replaced by fibrotic tissue, scarring and regenerative nodules, leading to liver malfunction.^1^, ^2^ It is often caused by chronic liver injury as a result of various aetiologies, including viral hepatitis, autoimmune liver diseases, alcoholic liver injury and non‐alcoholic fatty liver disease, and ultimately results in liver cirrhosis and hepatocellular carcinoma (HCC).^3^, ^4^

Activation of hepatic stellate cells (HSCs) is commonly recognized as a key step in hepatic fibrogenesis.^5^ In normal liver tissue, quiescent HSCs store vitamin A to maintain retinoid homeostasis.^6^ However, persistent liver injury can cause HSCs to acquire an activated phenotype and transdifferentiate into myofibroblast‐like cells that synthesize extracellular matrix components and produce the profibrogenic mediators.^7^ Thus, suppression of HSC activation appears to be a powerful therapeutic strategy for liver fibrosis development.

Inhibition of tyrosine kinases have attracted attention to therapeutically reduce fibrogenesis around for more than 20 years, and several tyrosine kinases have been recognized to regulate the activation of HSCs.^8^ For example, platelet‐derived growth factor receptor (PDGFR) and vascular endothelial growth factor receptor (VEGFR) were markedly increased during the development of liver fibrosis^9^ and were shown to activate multiple downstream signalling pathways, such as MEK/ERK and PI3K/Akt pathways, during HSC activation.^10^ These critical roles of tyrosine kinases in hepatic fibrogenesis have suggested the possibility that inhibition of tyrosine kinases could be beneficial as antifibrotic treatment.^11^ Several molecular targeted agents (MTAs) including tyrosine kinase inhibitors could exert efficient suppressive effects on proliferation, chemotaxis and collagen synthesis pathways in HSCs.^12^ Furthermore, beneficial outcomes by MTAs in liver fibrosis have been investigated in preclinical experimental animal models.^13^ For example, sorafenib, a MTA used to treat advanced HCC by targeting the Raf/ERK, VEGFR and PDGFRβ pathways, has been reported to show antifibrotic effects with inhibition of HSC activation in several experimental fibrotic rodent models.^14^ Moreover, early clinical trials showed that cirrhotic patients who received treatment with sorafenib exhibited a significant improvement of portal hypertension.^15^

Alterations to the hepatic vasculature have also been defined as key components in the process of liver fibrogenesis.^16^ Emerging evidence has indicated that aberrant architecture of micro‐vessels exacerbates portal hypertension and liver fibrosis progression.^17^ In parallel with sinusoidal capillarization, intrahepatic angiogenesis triggering shunt formation leads to increased portal vascular resistance and decreased effective parenchymal perfusion.^18^, ^19^ VEGFRs are the most crucial tyrosine kinases that participate in angiogenesis during liver fibrosis development, and studies have suggested that VEGFR‐targeting treatment using MTAs significantly attenuates liver fibrosis progression and decreases sinusoidal capillarization.^20^, ^21^

Lenvatinib is currently clinically approved in Japan, the United States and the European Union for use as first‐line treatment of unresectable HCC.^22^ It is an oral, small‐molecule MTA that targets VEGFR1‐3, fibroblast growth factor (FGF) receptor (FGFR)1‐4, PDGFRα/β, KIT and RET, and differs from sorafenib in that it targets FGF signalling pathways in HCC.^23^, ^24^ Lenvatinib demonstrated similar rates of severe toxicity and delayed decline in health‐related quality of life compared with sorafenib.^25^ These pharmacological targets of lenvatinib highlight its potential use as an antifibrotic agent, similar to sorafenib.

The present study examined the preventive effects of lenvatinib on in vitro activation of HSCs and in vivo liver fibrosis development and intrahepatic angiogenesis in CCl4‐induced rat fibrotic models.

## 2 MATERIALS AND METHODS

### 2.1 Cell culture

The human HSC line, LX‐2, was purchased from Merck. The cells were washed, suspended in Dulbecco's modified Eagle's medium (DMEM) (Nacalai tesque) supplemented with 10% foetal bovine serum (FBS) (Gibco) and antibiotics (1% penicillin and streptomycin), plated on 24‐well plastic culture dishes and incubated at 37°C in a 5% CO_2_ air environment.^26^ LX‐2 cells have been authenticated using Short Tandem Repeat profiling within the last 3 years. Mycoplasma testing was performed with MycoProbe^®^ Mycoplasma Detection Kit (R&D Systems, Inc) according to the manufacturer's protocol. For some assays, cells were incubated with recombinant transforming growth factor‐β1 (TGF‐β1) (Sigma‐Aldrich), or PDGF‐BB (Sigma‐Aldrich) or basic FGF (bFGF) (R&D Systems) with or without different concentrations of lenvatinib (ChemScene).

### 2.2 Cell viability assay

LX‐2 cells were seeded in 96‐well plates with DMEM which included 10% FBS for 24 hours. Then, the cells were exposed to different concentration of lenvatinib (0‐400 nmol/L) for 24 hours. The Premix WST‐1 Cell Proliferation Assay system (Takara Bio) was used to assess cell viability according to the manufacturer's protocol.

### 2.3 Cell proliferation assay

LX‐2 cells were seeded in 96‐well plates with DMEM which included 1% FBS for 24 hours. Then, the cells were exposed to different concentrations of PDGF‐BB (0‐1000 ng/mL) or bFGF (0‐60 ng/mL) for 24 hours or lenvatinib (0‐200 nmol/L) for 24, 48 or 72 hours. Moreover, for other set of experiments, the cells were pre‐treated with PDGF‐BB (50 ng/mL) or bFGF (10 ng/mL) for 2 hours and then were treated with lenvatinib (0‐400 nmol/L) for 24 hours. The BrdU Cell Proliferation ELISA (Cosmo Bio, Tokyo, Japan) was used to evaluate cell proliferation according to the manufacturer's protocol.

### 2.4 Measurement of cleaved caspase‐3 and cleaved PARP

To assess in vitro cell apoptosis, cleaved caspase‐3 and cleaved Poly (ADP‐ribose) polymerase (PARP) 1 concentrations in cell extract from LX‐2 cells were measured using Human Cleaved Caspase‐3 (Asp175) ELISA and Human Cleaved PARP1 ELISA kit (Abcam, Cambridge, UK), respectively, according to the manufacturer's instructions. A total of 1 × 10^6^ LX‐2 cells were treated with lenvatinib (0‐200 nmol/L) for 24 hours following overnight starvation.

### 2.5 Cell chemotaxis assay

Cell migration of LX‐2 cells was determined using the CytoSelect 24‐Well Cell Migration Assay (8 µm, Colorimetric Format) (Cell Biolabs, Inc, San Diego, CA, USA) according the manufacturer's instructions. A total of 1 × 10^6^ LX‐2 cells were pre‐treated with PDGF‐BB (50 ng/mL) for 2 h and then were treated with lenvatinib (0‐100 nmol/L) for 6 hours following overnight starvation.

### 2.6 Measurement of VEGFA levels

VEGFA concentration in the cultured media from LX‐2 cells was measured using RayBio Human VEGFA ELISA Kit (RayBiotech, Inc) according to the manufacturer's instructions. A total of 1 × 10^6^ LX‐2 cells were pre‐treated with PDGF‐BB (50 ng/mL) for 2 hours and then were treated with lenvatinib (0‐100 nmol/L) for 6 hours following overnight starvation.

### 2.7 Protein extraction and Western blotting

Proteins were extracted from 10^6^ cultured LX‐2 cells. For this purpose, T‐PER Tissue Protein Extraction Reagent as lysis buffer supplemented with proteinase and phosphatase inhibitors (Thermo Fisher Scientific) was used. The protein concentration was measured by protein assay (Bio‐Rad), and all samples were normalized to 100 μg. Western blotting was performed as described previously.^27^ The membranes were incubated overnight with antibodies against ERK1/2 (#9102, Cell Signaling Technology), phospho‐ERK1/2 (#4370S), Akt (#4691), phospho‐Akt (#4060), SMAD2/3 (#3102), phospho‐SMAD2/3 (#8828), PDGFRα (#3174), phospho‐PDGFα (Tyr1018) (#4547), PDGFRβ (#3169), phopho‐PDGFRβ (Tyr751) (#3166) and β‐actin (#4967). Densitometric analysis was performed with ImageJ software version 64 (National Institutes of Health).

### 2.8 Animals and experimental protocol

Six‐week‐old male Fisher 344 rats (Japan SLC) were treated twice a week for eight weeks with intraperitoneal injections of 0.5 mL/kg chronic carbon tetrachloride (CCl4) (diluted 1:10, Nacalai Tesque) or corn oil only as described previously.^28^ After 2 weeks of initial injections with CCl4 or corn oil, the administration of vehicle or lenvatinib was started. First, to optimize in vivo dose of lenvatinib, we administered the different doses of lenvatinib (0.4, 0.8, 1.2, 1.6, 3.2, 6.4 and 9.6 mg/kg) to CCl4‐mediated rats (n = 5) based on the previous reports to evaluate its anticancer effect in HCC xenograft model.^29^, ^30^ Next, the rats were divided into four groups (n = 10) according to treatment as follows: corn oil injections and vehicle administration (C/O group); CCl4 injections and vehicle administration (CCl4 group); CCl4 injections and low‐dose (0.4 mg/kg) lenvatinib (Ld group); and CCl4 injections and high‐dose (0.8 mg/kg) lenvatinib (Hd group). Because of incomplete water solubility of lenvatinib agent, we used water containing 10% carboxymethyl cellulose to suspend lenvatinib for oral administration to rats. Rats in the Ld and Hd groups received oral administration of lenvatinib by gavage daily throughout the experimental period. Water containing 10% carboxymethyl cellulose was given as vehicle. Rats were killed at the end of the eight‐week experimental period, then body and liver weights were measured and blood was collected from the aorta to measure serum levels of aspartate transaminase (AST), alanine transaminase (ALT), albumin (Alb), alkaline phosphatase (ALP) and bilirubin. Liver specimens were collected and immediately fixed in neutral‐buffered formalin. All animal procedures were performed in compliance with the recommendations of the Guide for Care and Use of Laboratory Animals of the National Research Council, and the study was approved by the ethics committee of Nara Medical University, Kashihara, Japan (No. 12585).

### 2.9 Histological and immunohistochemical analyses

Liver specimens were fixed in 10% formalin and embedded in paraffin. Sections of 5‐μm thickness were stained with haematoxylin and eosin (H&E) and Sirius‐Red. Primary antibodies, including alpha‐smooth muscle actin (α‐SMA) (ab124964, AbCam), CD34 (ab81289, AbCam), Desmin (413651; Nichirei Biosciences) and GFAP (ab7260, Abcam), were used, and staining was performed according to the manufacturer's instructions. A goat anti‐rabbit biotinylated secondary antibody was used and visualized using a horseradish peroxidase (HRP)‐conjugated ABC system (Vector Laboratories). DAB was used as the chromogen. Immunofluorescence for glutamine synthetase (GS) (ab176562, AbCam) and Ki67 (ab15580, AbCam) was performed on paraffin‐embedded intestinal sections using monoclonal antibodies against GS to assess hepatocyte expression and polyclonal antibodies against Ki67 to assess cell proliferation. Detection of the primary antibodies was performed with Alexa Fluor‐conjugated secondary antibodies (Invitrogen). Images were captured using a BX53 (Olympus) for histology and immunohistochemistry and a BZ‐X700 (Keyence) for immunofluorescence. Semi‐quantitative analysis was performed with ImageJ software version 64.

### 2.10 RNA extraction and reverse transcription‐quantitative polymerase chain reaction (RT‐qPCR)

Total RNA was isolated from liver tissues and 10^6^ cultured LX‐2 cells using the RNeasy Mini Kit (Qiagen). The resulting RNA concentrations were determined using a NanoDropTM 2000c Spectrophotometer (Thermo Fisher Scientific Inc). High‐capacity RNA‐to‐cDNA kit (Applied Biosystems) was used for reverse transcription to generate cDNA. Quantitative RT‐PCR (qRT‐PCR) was performed with the primer pairs described in Table S1 using a SYBR™ Green PCR Master Mix (Applied Biosystems) and an Applied Biosystems StepOnePlus™ Real‐Time PCR^®^ system (Applied Biosystems). Relative expression levels were normalized to *GAPDH/Gapdh* expression and estimated using the 2^−ΔΔCT^ method and presented as fold changes relative to controls.

### 2.11 Statistical analyses

Statistical analyses were performed with Prism, version 9 (GraphPad Software). Data are expressed as the mean ± standard deviation. Statistical variance between each experimental group was analysed using an analysis of variance test. Bartlett's test was used to determine homogeneity of variances. All tests were two‐tailed, and *P*‐values < 0.05 were considered statistically significant. We calculated the overall survival of rats in vivo experiments for lenvatinib dose optimization using the Kaplan‐Meier method. Analyses were conducted using EZR (Saitama Medical Center, Jichi Medical University), a graphical user interface of R version 2.13.0 (The R Foundation for Statistical Computing) and a modified version of R commander (version 1.6‐3) that includes statistical functions that are frequently used in biostatistics.^31^

## 3 RESULTS

### 3.1 Lenvatinib suppressed the proliferative capacity and TGF‐β1‐induced activation of human HSCs

**FIGURE 1:**
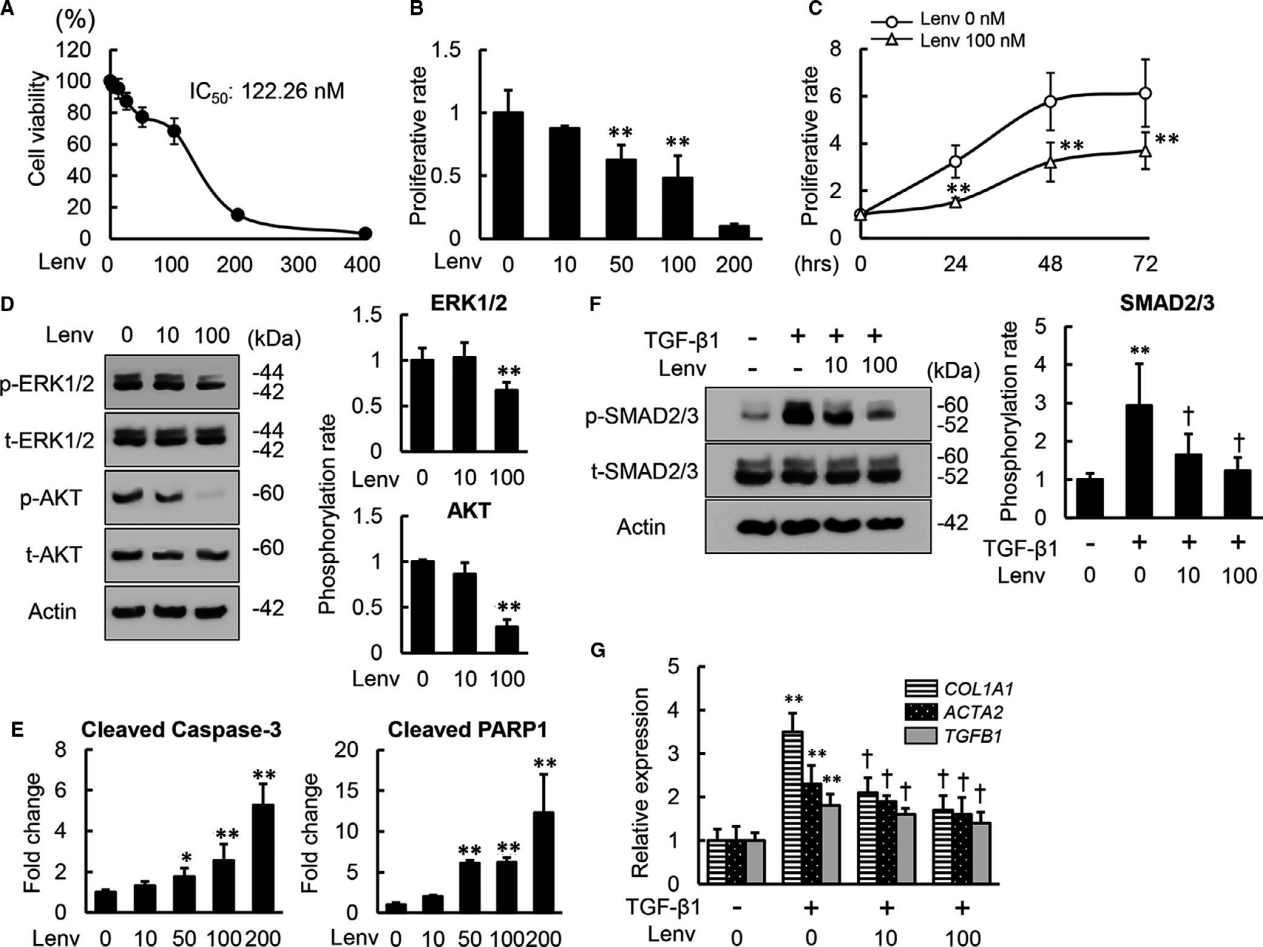
Effects of lenvatinib on in vitro proliferation, apoptosis and TGF‐β1‐induced activation of LX‐2 cells. A, Cell viability of LX‐2 incubated with lenvatinib (Lenv) (0‐400 nmol/L) for 24 h by WST‐1 assay. Cell viability was indicated as ratio to the value in the group of Lenv (0 nmol/L). B, Cell proliferation of LX‐2 incubated with Lenv (0‐200 nmol/L) for 24 h by BrdU assay. Cell proliferation was indicated as fold changes to the value in the group of Lenv (0 nmol/L). C, Time‐dependent effects of Lenv on cell proliferation in LX‐2 cells. The cells were cultured for 24, 48 and 72 h at 0 or 100 nmol/L of Lenv. D, Western blots (WBs) of whole cell lysates for total‐ and phospho‐ERK1/2 and AKT on LX‐2 cultured with Lenv (0‐100 nmol/L) for 6 h. Semi‐quantification of phosphorylation rate as ratio of p‐ERK1/2 to t‐ERK1/2 and p‐AKT to t‐AKT, respectively. E, The levels of cleaved caspase‐3 and cleaved PARP1 in LX‐2 cell culture extract assessed by ELISA. The levels were indicated as fold changes to the value in the group of Lenv (0 nmol/L). F, WBs of whole cell lysates for total‐ and phospho‐SMAD2/3 on LX‐2 cultured with Lenv (0‐100 nmol/L) for 3 h. Semi‐quantification of phosphorylation rate as ratio of p‐SMAD2/3 to t‐SMAD2/3. G, Relative mRNA expression levels of profibrogenic markers in LX‐2 cells. The mRNA expression levels were measured by qRT‐PCR, and *GAPDH* was used as internal control. Quantitative values are relatively indicated as fold changes to the values of non‐treatment groups. Actin was used as the loading control for WBs (D and F). Cells were pre‐treated with TGF‐β1 (10 ng/mL) 3 h before Lenv treatment (F and G). Data are mean ± SD (n = 3 independent experiments with n = 12 (A‐C,G) n = 3 (D, F) or n = 6 (E) samples per condition). *P < 0.05; **P < 0.01, indicating a significant difference compared with non‐treatment groups (B‐G). ^†^ *P* < 0.05, indicating a significant difference compared with TGF‐β1(+)/Lenv(−) groups (F and G)

Initially, we examined the impact of lenvatinib at different doses on cell viability of LX‐2 to optimize the concentrations of lenvatinib used for in vitro studies. As shown in Figure 1A, lenvatinib efficiently attenuated LX‐2 cell viability and 122.26 nmol/L was calculated as 50% inhibition concentration. Based on the optimal range of doses, we performed the following in vitro experiments.

The results of the BrdU assay showed that lenvatinib efficiently inhibited growth of LX‐2 cells cultured under normal conditions within the optimal doses (50 and 100 nmol/L) for 24 hours (Figure 1B). Time‐course assay confirmed that the antiproliferative effects of lenvatinib (100 nmol/L) persisted for at least for 72 hours (Figure 1C). Consistent with the suppression of cell proliferation, phosphorylation of ERK1/2 and AKT were attenuated in lenvatinib‐treated LX‐2 cells (Figure 1D). Moreover, treatment with lenvatinib dose‐dependently increased the levels of cleaved caspase‐3 and cleaved PARP1 in LX‐2 cell culture extract, indicating that lenvatinib has a potential to induce apoptosis of LX‐2 cells (Figure 1E). We next evaluated whether lenvatinib could exert antifibrogenic effects in activated HSCs by regulating the TGF‐β/Smad signalling pathway. Interestingly, TGF‐β1‐stimulated phosphorylation of Smad 2/3 was attenuated by treatment with lenvatinib of LX‐2 cells (Figure 1F). In accordance with interference of Smad 2/3 activation, lenvatinib significantly reduced mRNA expression levels of *COL1A1*, *ACTA2* and *TGFB1*, which was up‐regulated by TGF‐β1 stimulation (Figure 1G).

### 3.2 Lenvatinib inhibited PDGF‐stimulated proliferation, chemotaxis and VEGF production in human HSCs

**FIGURE 2:**
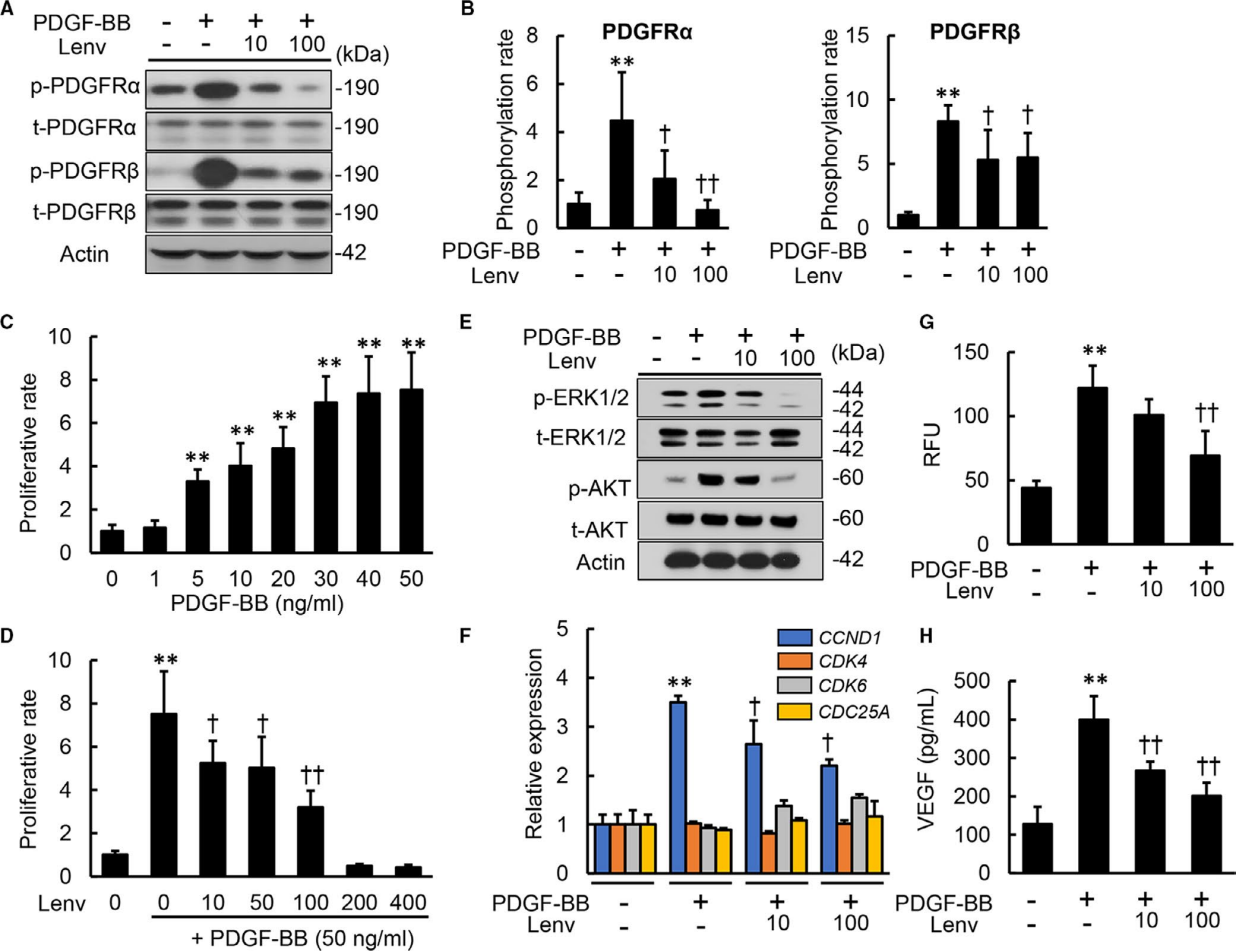
Effects of lenvatinib on in vitro PDGF signalling pathway in LX‐2 cells. A, Western blots (WBs) of whole cell lysates for the phosphorylation of PDGFRα and PDGFRβ on LX‐2 cultured with Lenv (0‐100 nmol/L) for 2 h. B, Semi‐quantification of phosphorylation rate in PDGFRα and PDGFRβ related to Figure 2A. C, Cell proliferation of LX‐2 cells stimulated by PDGF‐BB (0‐50 ng/mL) for 24 h. D, Cell proliferation of LX‐2 cells incubated with Lenv (0‐400 nmol/L) for 24 h. E, WBs of whole cell lysates for total‐ and phospho‐ERK1/2 and AKT on LX‐2 cultured with Lenv (0‐100 nmol/L) for 6 h. F, Relative mRNA expression levels of cell cycle‐related markers in LX‐2 incubated with Lenv (0‐100 nmol/L) for 12 h. The mRNA expression levels were measured by qRT‐PCR, and *GAPDH* was used as internal control. G, Cell migration of LX‐2 incubated with Lenv (0‐100 nmol/L) for 6 h. Relative fluorescence units (RFU) was determined as cell migration. H, VEGFA levels in LX‐2‐cultured media. Actin was used as the loading control for WBs (A and E). Cells were pre‐treated with PDGF‐BB (50 ng/mL) 2 h before Lenv treatment (A,B,D‐H). Data are mean ± SD (n = 3 independent experiments with n = 3 (B), n = 12 (C,D,F) or n = 6 (G,H) samples per condition). Quantitative values are relatively indicated as fold changes to the values of non‐treatment groups (B‐D,F). *P < 0.05; **P < 0.01, indicating a significant difference compared with non‐treatment groups (B‐D, F‐H). ^†^ *P* < 0.05, ^††^ *P* < 0.01, indicating a significant difference compared with PDGF‐BB(+)/Lenv(−) groups (B,D,F‐H)

As lenvatinib is known to pharmacologically inhibit PDGFR, we next analysed its effects on PDGF‐induced phenotypic changes in human HSCs. Treatment of LX‐2 cells with lenvatinib (10 and 100 nmol/L) down‐regulated protein levels of phospho‐PDGFRα, which was induced by PDGF‐BB stimulation (Figure 2A). Remarkably, PDGF‐BB‐induced up‐regulation of phospho‐PDGFRβ were also reduced by treatment with lenvatinib (Figure 2A). The phosphorylation rates (phosphorylated/total) of both PDGFRα and PDGFRβ were significantly decreased in lenvatinib‐treated LX‐2 cells (Figure 2B). These findings indicated that lenvatinib efficiently inhibits phosphorylation of PDGFRβ as well as PDGFRα in human HSCs. Next, we assessed the effects of lenvatinib on HSC growth by regulating PDGFR signalling. PDGF‐BB (1‐50 ng/mL) dose‐dependently induced LX‐2 proliferation (Figure 2C). In agreement with the inhibitory effects of lenvatinib on PDGFR, effective concentrations (10‐100 nmol/L) of lenvatinib substantially suppressed PDGF‐BB (50 ng/mL)‐stimulated proliferation of LX‐2 cells (Figure 2D). These antiproliferative effects of lenvatinib were involved in the interference of PDGF‐BB‐induced phosphorylation of ERK 1/2 and AKT via blockade of the PDGFR signalling process in LX‐2 cells (Figure 2E). The effects of lenvatinib on cell cycle distribution we examined by analysing the molecular basis of cell cycle arrest. Quantitative PCR analysis revealed that lenvatinib significantly reduced PDGF‐BB‐induced overexpression of cyclin D1 (*CCND1*), which plays a pivotal role in regulating the G_1–S_ phase transition in LX‐2 cells (Figure 2F). PDGF has been shown to promote chemotaxis of HSCs during the profibrogenic process. Thus, we next assessed the effects of lenvatinib on cell migration of HSCs. Lenvatinib decreased the PDGF‐BB‐mediated migratory potential of LX‐2 cells (Figure 2G). Previous studies have shown that PDGF could increase VEGF protein levels in activated HSCs. Remarkably, lenvatinib‐mediated PDGFR inhibition decreased VEGF production in LX‐2 cells (Figure 2H).

### 3.3 Lenvatinib suppressed bFGF‐stimulated cell growth in human HSCs

**FIGURE 3:**
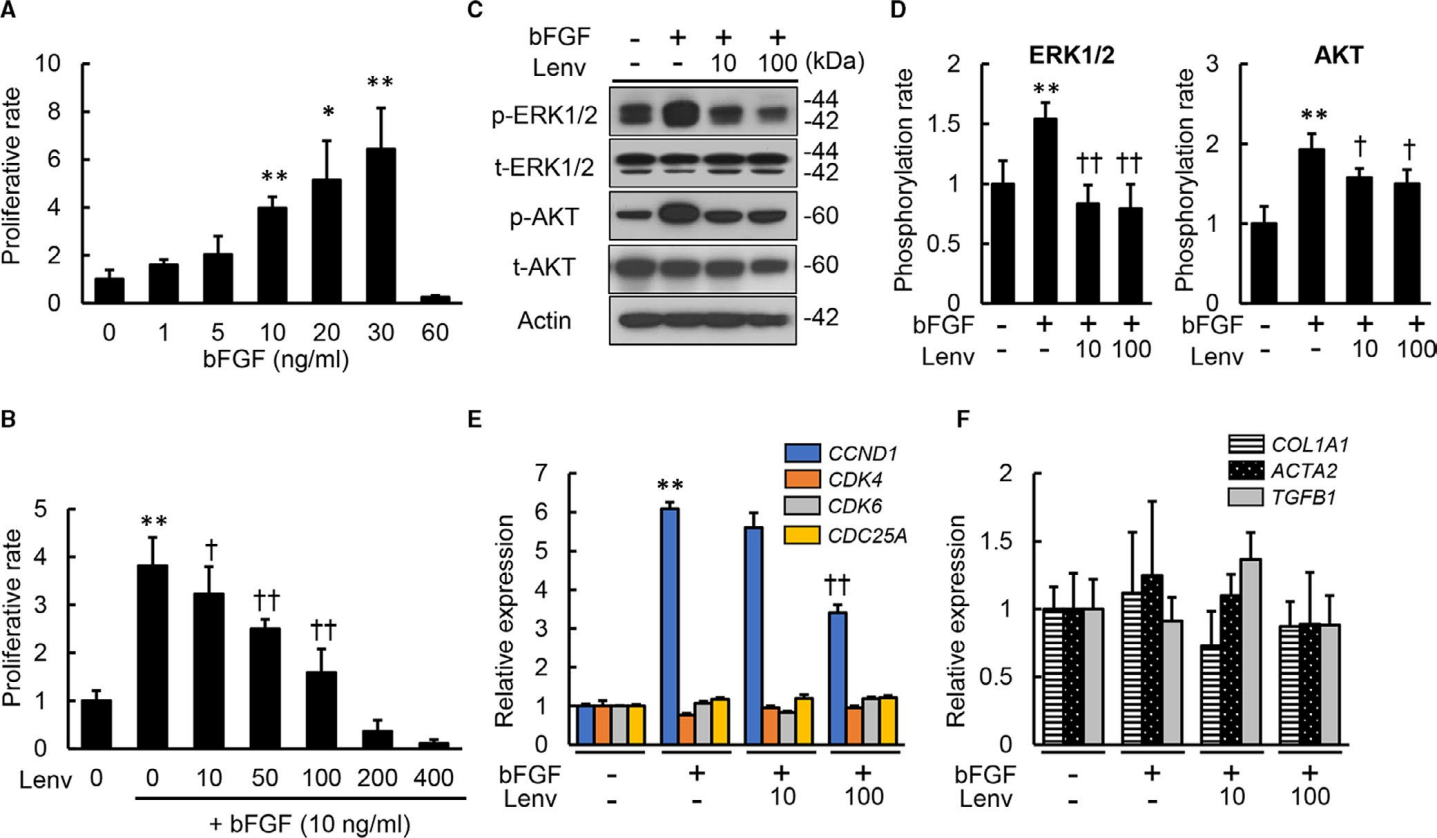
Effects of lenvatinib on in vitro bFGF‐stimulated LX‐2 cell growth. A, Cell proliferation of LX‐2 cells stimulated by bFGF (0‐60 ng/mL) for 24 h. B, Cell proliferation of LX‐2 cells incubated with Lenv (0‐400 nmol/L). C, Western blots of whole cell lysates from LX‐2 incubated with Lenv (0‐100 nmol/L) for the phosphorylation of ERK1/2 and AKT. Actin was used as internal control. D, Semi‐quantification of phosphorylation rate in ERK1/2 and AKT related to Figure 3C. (E and F) Relative mRNA expression levels of (E) cell cycle‐related markers and (F) profibrogenic markers in LX‐2 co‐incubated with bFGF (10 ng/mL) and Lenv (0‐100 nmol/L). The mRNA expression levels were measured by qRT‐PCR, and *GAPDH* was used as internal control. Cells were pre‐treated with bFGF (10 ng/mL) 2 h before Lenv treatment (B‐F). Data are mean ± SD (n = 3 independent experiments with n = 12 (A,B,E,F) or n = 3 (D) samples per condition). Quantitative values are relatively indicated as fold changes to the values of non‐treatment groups. *P < 0.05; **P < 0.01, indicating a significant difference compared with non‐treatment groups (A,B,D‐F). ^†^ *P* < 0.05, ^††^ *P* < 0.01, indicating a significant difference compared with bFGF(+)/Lenv(‐) groups (A,B,D‐F)

Lenvatinib also inhibits FGFRs; therefore, we next evaluated its effects on bFGF‐induced proliferation of HSCs. We confirmed the biological activity of bFGF induction of cell proliferation in LX‐2 cells (Figure 3A). Lenvatinib exerted antiproliferative effects on bFGF‐stimulated LX‐2 cells in a similar manner to the effects on PDGF‐stimulated cells (Figure 3B). Similarly, lenvatinib inhibited phosphorylation of both ERK1/2 and AKT in LX‐2 cells treated with bFGF (Figure 3C,D) and also significantly suppressed overexpression of *CCND1* induced by bFGF treatment (Figure 3E). On the other hand, mRNA expression levels of profibrogenic markers were unchanged in bFGF‐stimulated LX‐2 cells, and treatment with lenvatinib did not affect their expression (Figure 3F).

### 3.4 Lenvatinib attenuated hepatic fibrosis development in CCl4‐treated rats

**FIGURE 4:**
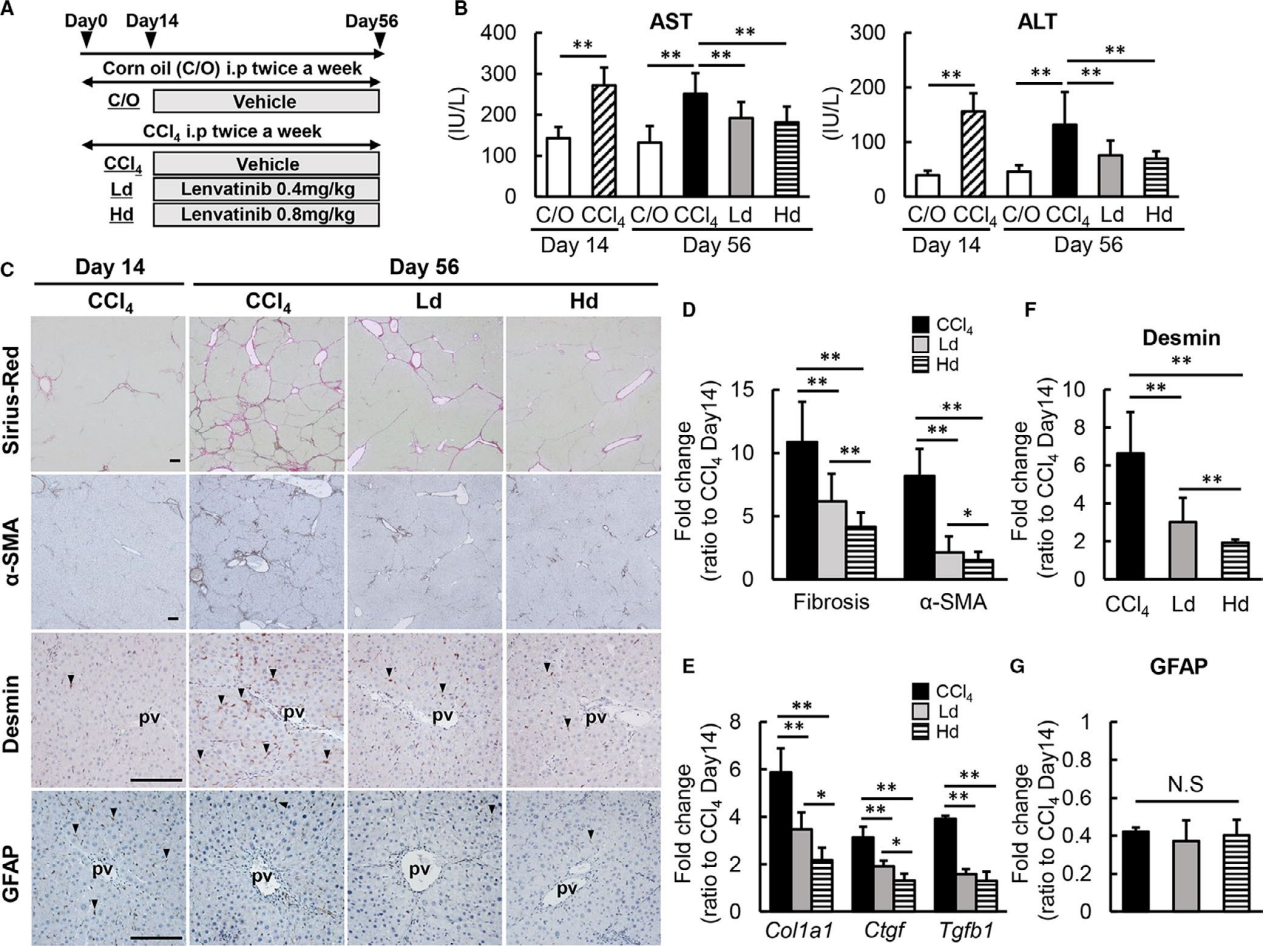
Effects of lenvatinib on CCl4‐induced liver fibrosis in rats. A, Experimental protocol. B, Serum levels of aspartate transaminase (AST) and alanine aminotransferase (ALT) in the experimental groups. C, Representative microphotographs of Sirius‐Red, α‐SMA, Desmin and GFAP staining in the experimental groups. Scale bar; 100 μm. pv; portal vein. D, Semi‐quantitation of Sirius‐Red‐stained fibrotic area and α‐SMA immuno‐positive area in high‐power field (HPF) by ImageJ software. E, Relative mRNA expression levels of *Col1A1, Ctgf* and *Tgfb1* in the experimental groups. The mRNA expression levels were measured by qRT‐PCR, and *Gapdh* was used as internal control. (F,G) Semi‐quantitation of Desmin (F) and GFAP (G) immuno‐positive area in high‐power field (HPF) by ImageJ software. C/O, Corn oil‐administered group; CCl4, CCl4 with vehicle‐administered group, Ld; CCl4 with low dose of lenvatinib‐administered group, Hd; CCl4 with high dose of lenvatinib‐administered group. Data are mean ± SD (n = 10). Histochemical quantitative analyses included five fields per section (D,F,G). Quantitative values are relatively indicated as fold changes to the values of CCl4 at day 14 (D‐G). *P < 0.05; **P < 0.01, indicating a significant difference between groups (B,D‐G). NS, not significant

Given the cell‐based actions of lenvatinib on HSCs, we examined the effect on hepatic fibrosis development using a CCl4‐induced liver fibrosis rat model (Figure 4A). Initially, we optimized the dose of lenvatinib by evaluating the overall survival of rats receiving the different doses (0.4, 0.8, 1.2, 1.6, 3.2, 6.4 and 9.6 mg/kg) to CCl4‐mediated rats. As shown in Figure S1A, the rats which were administered with more than 3.2 mg/kg were all dead within 4 weeks from start of lenvatinib administration as a result of the impairment of detoxification capacity to lenvatinib in rats by CCl4 administration. Moreover, 80% of rats which were administered with 1.2 or 1.6 mg/kg were also dead within 4 weeks from start of administration. By contrast, all of rats which were administered with 0.4 or 0.8 mg/kg were alive at the end of experiment. Consequently, we defined that these doses were suitable for CCl4‐mediated rats. Systemic analysis showed that body and liver weights remained unchanged by oral administration of lenvatinib at both low (0.4 mg/kg) and high (0.8 mg /kg) doses in this experimental model, indicating that these doses led to low toxicity (Figure S1B). Serum levels of AST and ALT were not elevated, but declined following treatment with lenvatinib in CCl4‐treated rats (Figure 4B). These results suggest that lenvatinib shows hepatoprotective properties against chemically induced liver injury.

The fibrotic areas stained by Sirius‐Red were mildly increased by CCl4 administration for two weeks at the start of the lenvatinib treatments (Figure S2). Continuous administration of CCl4 for another six weeks significantly developed liver fibrosis which were remarkably suppressed by following treatment with lenvatinib (Figure 4C). Semi‐quantitative analysis demonstrated that the fibrosis degree relative to the start of the lenvatinib treatment was suppressed by >50% in CCl4‐treated rats treated with lenvatinib, especially at higher doses (Figure 4D). Along with liver fibrosis, lenvatinib treatment attenuated the number of α‐SMA‐positive myofibroblasts which was increased in CCl4‐mediated rats (Figure S2, Figure 4C,D). These ameliorations in the fibrotic phenotypes coincided with reduced hepatic mRNA expressions of profibrotic genes, including *Col1a1, Tgfb1* and *Ctgf* (Figure 4E). Thereafter, we performed immunohistochemical analysis of Desmin and GFAP to assess the intrahepatic activation of HSCs. Desmin‐positive activated HSCs were decreased in lenvatinib‐treated groups (Figure 4C,F). On the other hand, GFAP‐positive quiescent HSCs were unchanged in lenvatinib‐treated groups (Figure 4C,G). These findings possibly suggest that lenvatinib‐mediated decrease in α‐SMA‐positive myofibroblasts is predominantly as a result of apoptosis of activated HSCs and is less relevant to reversal into quiescent phenotype.

### 3.5 Lenvatinib suppressed intrahepatic capillarization with decreased transcription of growth factors and their receptors in CCl4‐treated rats

**FIGURE 5:**
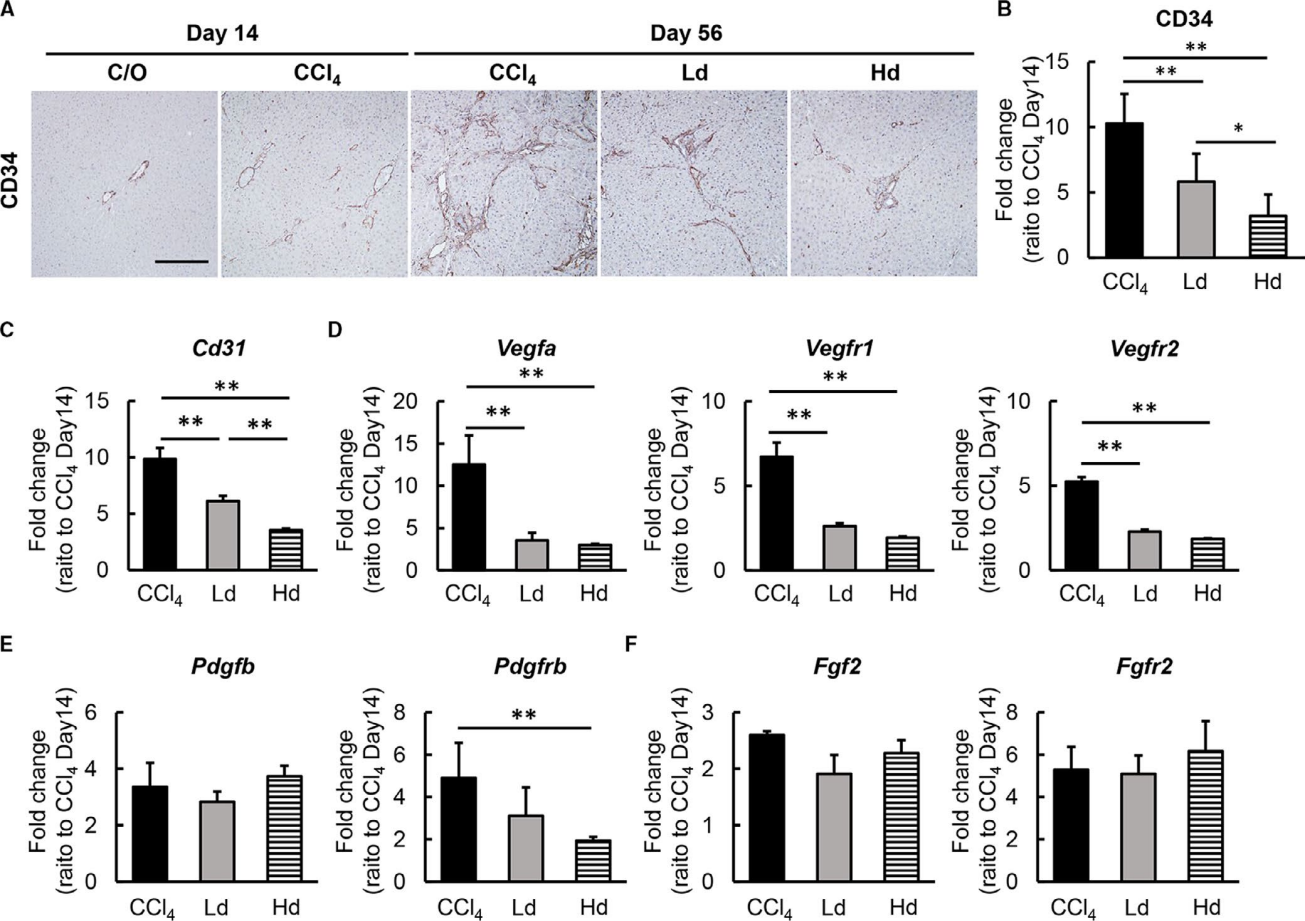
Effects of lenvatinib on intrahepatic capillarization and angiogenesis in CCl4‐induced fibrotic liver. A, Representative microphotographs of CD34 staining in the experimental groups. Scale bar; 50 μm. B, Semi‐quantitation of CD34‐positive vessels in the experimental groups in high‐power field (HPF) by ImageJ software. Quantitative analysis included five fields per section. (C‐F) Relative mRNA expression of (C) *Cd31*, (D) *Vegfa, Vegfr1* and *Vefgr2*, (E) *Pdgfb* and *Pdgfrb*, (F) *Fgf2* and *Fgfr* in the experimental groups. The mRNA expression levels were measured by qRT‐PCR, and *Gapdh* was used as internal control. C/O, Corn oil‐administered group; CCl4, CCl4 with vehicle‐administered group; Ld; CCl4 with low dose of lenvatinib‐administered group, Hd; CCl4 with high dose of lenvatinib‐administered group. Data are mean ± SD (n = 10). Quantitative values are relatively indicated as fold changes to the values of CCl4 at day 14 (B‐F). *P < 0.05; **P < 0.01, indicating a significant difference between groups (B‐F)

Intrahepatic capillarization is reported to exacerbate liver fibrosis development. Therefore, we also evaluated changes in angiogenic status to determine the relevance of antiangiogenic activity in lenvatinib on attenuated liver fibrosis in experimental rat livers. Newly formed CD34‐positive intrahepatic vessels were mildly increased by CCl4 administration for two weeks at the start of the lenvatinib treatment along with liver fibrosis (Figure 5A). Continuous administration of CCl4 for another six weeks progressively enhanced intrahepatic capillarization which were remarkably suppressed in lenvatinib‐mediated rats (Figure 5A). Semi‐quantitative analysis showed a profound reduction in the capillarization degree relative to the start of the lenvatinib treatment in the Hd group to <25% (Figure 5B). This finding coincided with the decreased hepatic mRNA levels of *Cd31* observed in both Ld and Hd groups (Figure 5C). As the major targets of lenvatinib, including VEGF/VEGFR, PDGF/PDGFR and FGF/FGFR, are known to act as angiogenic regulators, we next assessed hepatic mRNA expression of these growth factors and receptors. Hepatic mRNA levels of *Vegfa*, *Vegfr1* and *Vegfr2* were increased in accordance with CCl4‐induced liver fibrosis and were significantly reduced in response to treatment with lenvatinib (Figure 5D). Lenvatinib treatment also led to decreased mRNA levels of *Pdgfrb*, which was increased in CCl4‐induced fibrotic liver, whereas *Pdgfb* mRNA levels were not affected (Figure 5E). The mRNA levels of both *Fgf2* and *Fgfr* remained unchanged by treatment with lenvatinib (Figure 5F). These results suggest that the antiangiogenic effects of lenvatinib contribute to the amelioration of CCl4‐induced hepatic fibrosis development.

### 3.6 Lenvatinib treatment showed no detrimental effect on hepatocyte proliferation

**FIGURE 6:**
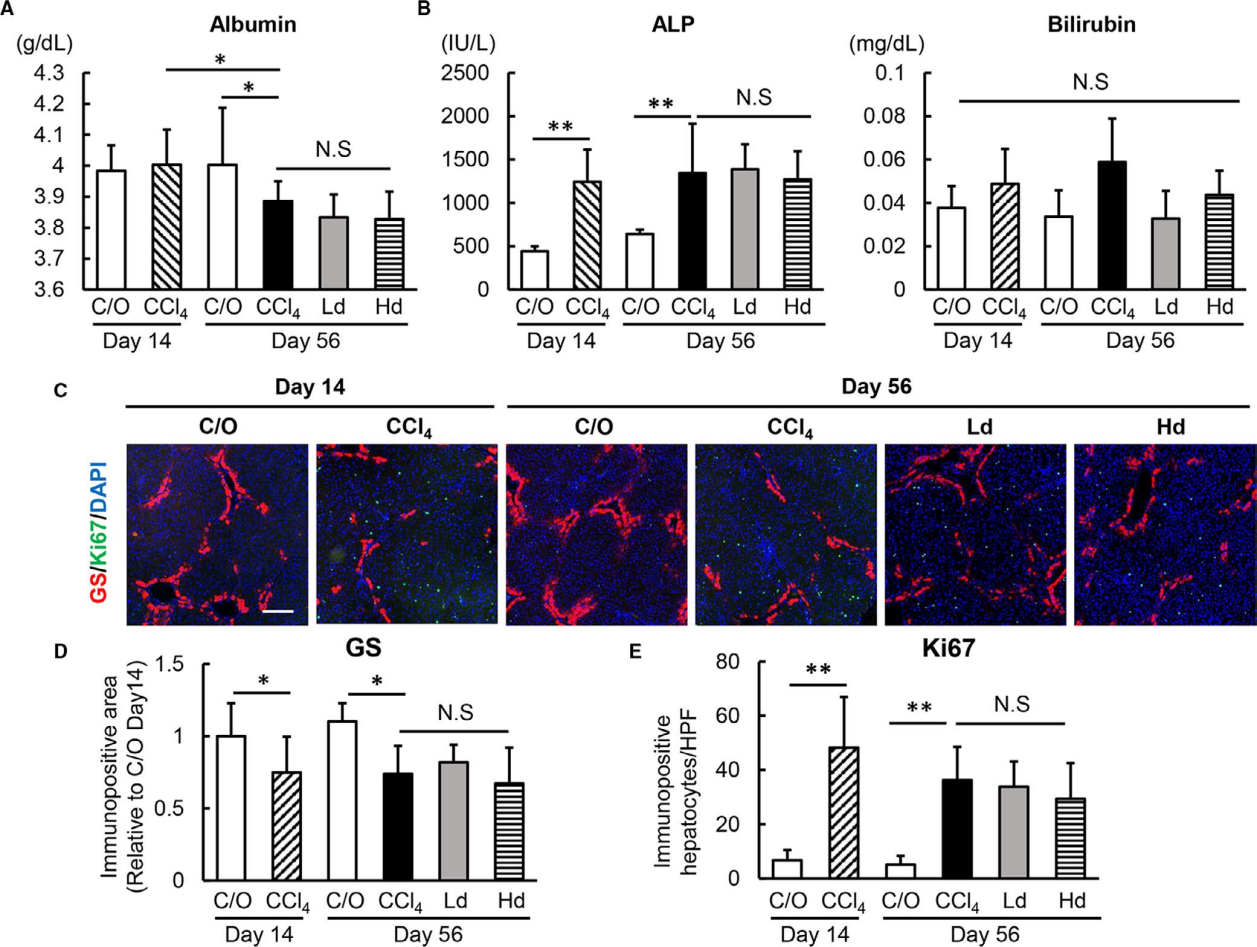
Effects of lenvatinib on hepatocytes in CCl4‐induced fibrotic liver. A, Serum levels of albumin; B, alkaline phosphatase (ALP) and bilirubin (B) in the experimental groups. C, Representative microphotographs of double‐immunofluorescence with GS/Ki67/DAPI staining in the experimental groups. Scale bar; 50 μm. (D,E) Semi‐quantitation of GS‐positive area (D) and Ki67‐positive cells (E) in high‐power field (HPF) by ImageJ software in the experimental groups. Quantitative values are relatively indicated as fold changes to the values of C/O at day 14 (D). The number of Ki67/DAPI‐positive hepatocytes were counted per 800 DAPI‐positive hepatocytes in HPF (E). Quantitative analyses included five fields per section. Ct, control; Ld, low dose of lenvatinib; Hd, high dose of lenvatinib; Veh; vehicle. Data are mean ± SD (n = 10). *P < 0.05; indicating a significant difference between groups (A,B,D and E). NS, not significant

Finally, we examined whether lenvatinib affected hepatocytes in pericentral zone 3 injured by CCl4 administration as well as proliferation of hepatocytes in periportal zones 1 and 2. Serum albumin levels were not changed by two weeks of CCl4 administration but were decreased by eight weeks administration. In CCl4‐treated rats, administration of lenvatinib at low and high doses did not alter serum albumin levels, indicating that these doses did not extensively damage hepatocytes (Figure 6A). Furthermore, treatment with lenvatinib did not change serum levels of ALP and bilirubin, indicating low biliary epithelial damage (Figure 6B). Moreover, immunostaining for GS demonstrated that the number of hepatocytes in pericentral zone 3 remained unchanged by treatment with lenvatinib compared with vehicle treatment in CCl4‐treated rats (Figure 6C,D). Additionally, the numbers of Ki67‐positive proliferative hepatocytes in zones 1 and 2 were not altered by lenvatinib‐treated rats compared with vehicle‐treated rats (Figure 6C,E). These results suggest low detrimental effects of lenvatinib on hepatic regeneration via proliferation of hepatocytes in CCl4‐induced liver injury.

## 4 DISCUSSION

The present study revealed that lenvatinib efficiently attenuated progression of CCl4‐induced liver fibrosis in rats. The observed antifibrotic effects mediated by lenvatinib may potentially be involved in several underlying mechanisms as a result of its pharmacological properties as a MTA.^32^

We confirmed the effects of lenvatinib on in vitro phenotypes of cultured HSCs. As with previous studies into the effects of sorafenib on HSCs, lenvatinib significantly suppressed cell proliferation of LX‐2 cells, along with inhibition of ERK1/2 and AKT signal transduction.^33^, ^34^ Moreover, our results showed that lenvatinib enhanced the cleavage of caspase‐3 and PARP in cultured LX‐2 cells suggesting the induction of apoptosis. Meanwhile, recent cell‐based studies have shown that lenvatinib does not affect cell apoptosis in human liver cancer cells.^30^ This discrepancy on apoptosis between cell types is an issue to be further addressed.

Interestingly, our results demonstrated that lenvatinib could inhibit TGF‐β‐mediated profibrogenic activities via the SMAD‐dependent signalling pathway in LX‐2 cells, although it did not exhibit antagonism against TGF‐β receptors. We suspect that this inhibitory effect is associated with blockade of PDGFR by lenvatinib. A recent study reported that PDGFRα could interact with SMAD‐dependent TGF‐β signalling during the process of HSC activation via both transcriptional and post‐transcriptional regulation.^35^, ^36^ In line with this, our in vitro data showed that lenvatinib significantly suppressed the PDGF‐BB‐stimulated PDGFRα phosphorylation in LX‐2. This result supports the possible effects of lenvatinib on the TGFβ/SMAD pathway; however, further molecular studies are required to clarify the mechanisms involved.

Our findings showed that lenvatinib also suppressed the PDGF‐BB‐stimulated phosphorylation of PDGFRβ as well as PDGFα in LX‐2 cells. Among the PDGF family members, PDGF‐BB is the most powerful in priming HSC proliferation, chemotaxis and phenotypic change to myofibroblast followed by collagen synthesis via predominantly PDGFRβ‐driven intracellular signalling.^37^, ^38^ In liver tissue obtained from patients with chronic liver diseases, expression of PDGF and its receptor subunits appears to be strictly correlated with the extent of fibrosis.^39^ Transgenic overexpression of PDGF‐BB in mice led to spontaneous development of liver fibrosis without up‐regulation of TGF‐β.^40^ This functional insight into the PDGF signalling pathway has led to an increased interest in the antifibrotic agents targeting this pathway. Our previous studies demonstrated that imatinib mesylate, a MTA used for patients with chronic myeloid leukaemia, acute lymphoblastic leukaemia and gastrointestinal stromal tumour, attenuated pig serum‐induced liver fibrosis development in rats by inhibiting PDGF signalling.^41^, ^42^ The results of the present study exhibited significant suppression of PDGF‐BB‐induced proliferation and chemotaxis in LX‐2 by lenvatinib, indicating its efficacious inhibition of PDGF/PDGFR could ameliorate the phenotypic changes of HSCs.

Moreover, we found that lenvatinib suppressed bFGF‐stimulated HSCs proliferation. There is conflicting evidence regarding the biological action of bFGF on HSC function and proliferation. Some studies have claimed that bFGF induces cell proliferation and up‐regulates α‐SMA expression in HSCs.^43^, ^44^, ^45^ Lin et al recently reported that bFGF promotes cell proliferation by inducing activation of the ERK signalling pathway and altering expression of cyclin D in primary rat HSCs.^45^ Nakamura et al also reported that bFGF showed mitogenic activity in LX‐2 cells, which was inhibited by cotreatment with brivanib, an ATP‐competitive inhibitor of FGFR, VEGFR and PDGFR.^28^ In contrast, Matsubara et al demonstrated that bFGF acts as a suppressor of profibrogenic activity in human HSCs by up‐regulating gene expression of CYGB, a reactive oxygen species scavenger.^46^ Pan et al suggested that low‐ and high‐molecular‐weight bFGF play opposing roles.^47^ In the present study, stimulation of bFGF did not alter mRNA levels of profibrogenic markers in LX‐2 cells, and lenvatinib did not affect the expression of these markers in bFGF‐treated LX‐2 cells. However, further studies are required to elucidate the functional mechanisms involved and the role of bFGF in HSCs using bFGF isoforms, as well as the effects of lenvatinib.

Chronic administration of lenvatinib significantly reduced intrahepatic angiogenesis along with attenuation of CCl4‐induced liver fibrosis in rats. In all lenvatinib‐treated groups, hepatic expression of VEGFR1 and VEGFR2 were markedly decreased compared with the vehicle‐treated group, in parallel with CD34‐positive neovascularization. These results indicate that lenvatinib may exert its effects on sinusoidal capillarization via the VEGFR signalling pathway. Furthermore, the present study elucidated that lenvatinib significantly decreased PDGF‐BB‐stimulated VEGFA production in LX‐2 cells and reduced mRNA levels of VEGFA in CCl4‐induced fibrotic livers. As PDGF is reported to stimulate HSCs to acquire an angiogenic phenotype via modification of HSC‐based vascular formation, these findings suggest that the antiangiogenic effects of lenvatinib can be attributed to PDGFR signalling blockade in HSCs as well as VEGFR signalling on sinusoidal endothelial cells.^48^, ^49^ The present results reinforce the functional connectivity between intrahepatic angiogenesis and liver fibrosis development. Moreover, these findings support paracrine signalling between sinusoidal endothelial cells as well as the orchestration of fibrogenesis, angiogenesis and portal hypertension by HSCs.^50^

The present study has several considerable limitations. As lenvatinib is a MTA used in the treatment of HCC, it is clinically administered to patients who tend to have established liver fibrosis.^51^ In the present study, oral administration of lenvatinib began two weeks after the initiation of CCl4‐induced liver fibrosis. This period is estimated as the point before the loss of hepatic functional reserve because lenvatinib is not available for patients with decompensated cirrhosis in the clinical settings. Further studies are required to evaluate the effect of lenvatinib in more advanced fibrotic models to verify whether it is clinically beneficial for liver fibrosis. Additionally, this study evaluated the effect of lenvatinib on in vivo liver fibrogenesis only in a single animal model. To reinforce translational impact, it is necessary to assess the effects in an additional model of liver fibrosis (eg a NASH model). Finally, results of this study found that administration of lenvatinib did not significantly affect in vivo proliferation of hepatocytes during CCl4‐induced chronic liver injury. This agent suppressed in vitro proliferation of human liver cancer cells, whereas we speculate that this potentially facilitates hepatic regeneration by inhibiting sinusoidal capillarization to some extent y.^52^ Thus, there may be a possibility that these opposing effects of lenvatinib on hepatocyte proliferation offset each other in the current model.

Collectively, the present study is the first to report that lenvatinib prevents liver fibrosis progression using an experimental model and that this effect is based on the suppression of HSCs proliferation, migration and profibrogenic activity as well as attenuation of sinusoidal capillarization. It is important to emphasize that these actions of lenvatinib were achieved using a pharmacological dose without toxic hepatocytes damage. Although lenvatinib has been approved for the treatment of HCC, our findings indicate that this drug could be used as a novel therapeutic option for liver fibrosis development.

## CONFLICT OF INTEREST

No potential conflicts of interest were disclosed by all authors.

## AUTHOR CONTRIBUTIONS

**Hiroyuki Ogawa:** Data curation (lead); Formal analysis (lead); Investigation (lead); Methodology (supporting); Writing‐original draft (lead). **Kosuke Kaji:** Conceptualization (lead); Data curation (equal); Methodology (supporting); Resources (equal); Supervision (supporting); Validation (supporting); Visualization (lead); Writing‐review & editing (lead). **Norihisa Nishimura:** Formal analysis (equal); Investigation (supporting); Writing‐review & editing (supporting). **Hirotetsu Takagi:** Investigation (supporting); Writing‐review & editing (supporting). **Koji Ishida:** Investigation (supporting); Writing‐review & editing (supporting). **Hiroaki Takaya:** Software (lead); Writing‐review & editing (supporting). **Hideto Kawaratani:** Formal analysis (supporting); Visualization (equal); Writing‐review & editing (supporting). **Kei Moriya:** Validation (lead); Writing‐review & editing (supporting). **Tadashi Namisaki:** Methodology (lead); Writing‐review & editing (supporting). **Takemi Akahane:** Supervision (equal); Writing‐review & editing (supporting). **Hitoshi Yoshiji:** Conceptualization (equal); Resources (lead); Supervision (lead); Writing‐review & editing (equal).

## Supporting information

Fig S1

Fig S2

Supplementary Material

## Data Availability

The data that support the findings of this study are available from the corresponding author upon reasonable request.
