# Supplementary figures and images for: Lenvatinib prevents liver fibrosis by inhibiting hepatic stellate cell activation and sinusoidal capillarization in experimental liver fibrosis
Source: J Cell Mol Med. 2021 Feb 20;25(8):4001–13. doi: 10.1111/jcmm.16363 (PMC8051749; doi:10.1111/jcmm.16363)

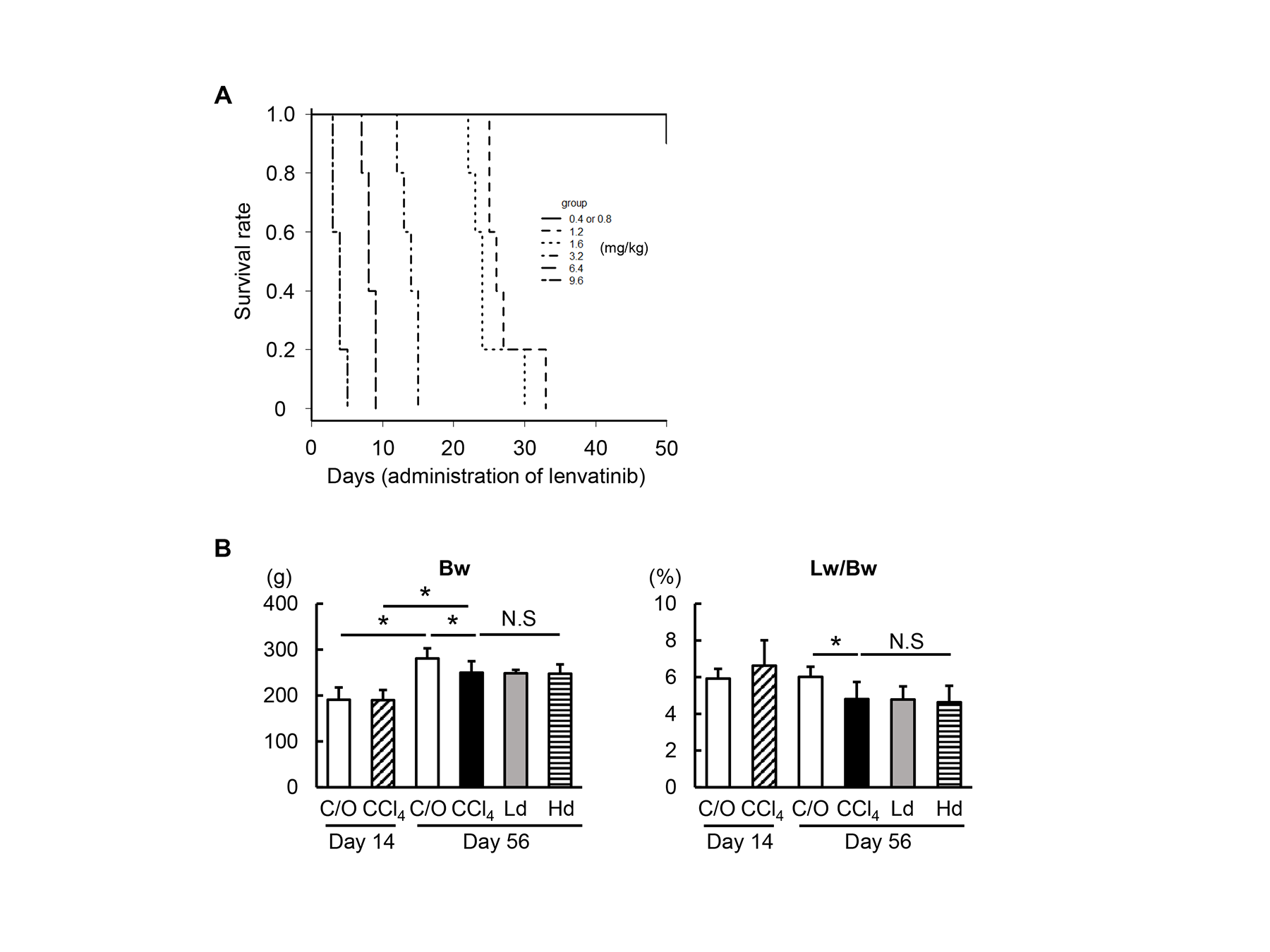

Supplement: Supplementary file 1 — Fig S1 [file JCMM-25-4001-s003.tif]

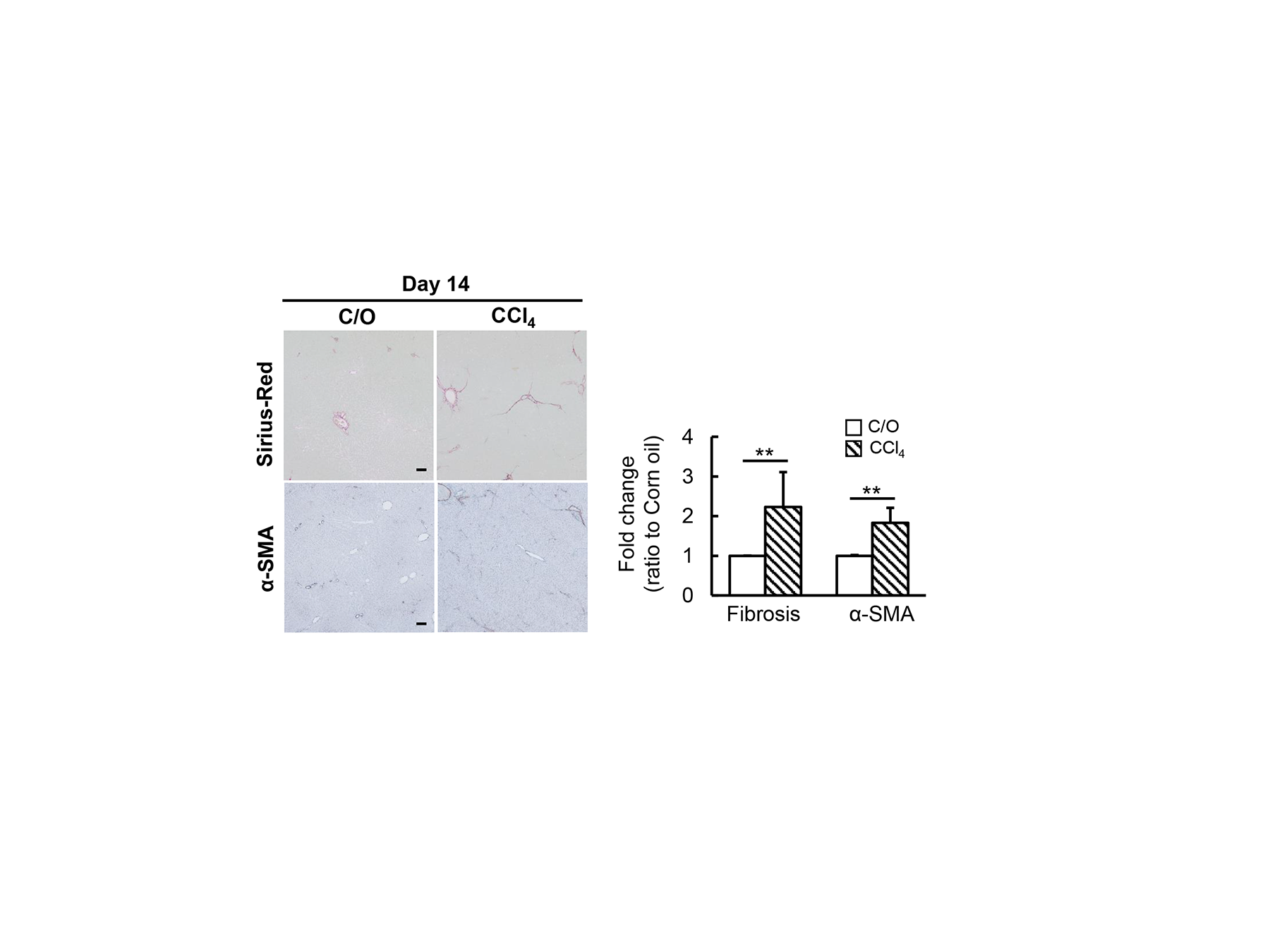

Supplement: Supplementary file 2 — Fig S2 [file JCMM-25-4001-s002.tif]
