# Supplementary material for: Lenvatinib prevents liver fibrosis by inhibiting hepatic stellate cell activation and sinusoidal capillarization in experimental liver fibrosis
Source: J Cell Mol Med. 2021 Feb 20;25(8):4001–13. doi: 10.1111/jcmm.16363 (PMC8051749; doi:10.1111/jcmm.16363)
Supplement: Supplementary file 3 — Supplementary Material [file JCMM-25-4001-s001.docx]

**Supplementary Materials**

**Lenvatinib prevents liver fibrosis progression and inhibits hepatic stellate cell activation and sinusoidal capillarization in experimental liver fibrosis**

Hiroyuki Ogawa, Kosuke Kaji, Norihisa Nishimura, Hirotetsu Takagi, Koji Ishida, Hiroaki Takaya, Hideto Kawaratani, Kei Moriya, Tadashi Namisaki, Takemi Akahane, Hitoshi Yoshiji

Department of Gastroenterology, Nara Medical University, Kashihara, Nara 634-8521, Japan

Address correspondence to:

Kosuke Kaji M.D., Ph.D.

Department of Gastroenterology, Nara Medical University

840 Shijo-cho, Kashihara, Nara 634-8521, Japan

E-mail: [kajik@naramed-u.ac.jp](mailto:kajik@naramed-u.ac.jp)

**1. Supplementary figure legend**

**2. Supplementary Table. 1**

**Supplementary figure legend**

**Supplementary Figure 1. In vivo dose optimization of lenvatinib and body and liver weight in CCl4-mediated rats.**

(A) Kaplan-Meier curves show the overall survival of CCl4-treated rats receiving administration of the different doses (0.4, 0.8, 1.2, 1,6, 3.2, 6.4 and 9.6 mg/kg) of lenvatinib. (B) Body weight (Bw) and ratio of liver weight to body weight (Lw/Bw) in the experimental groups at the end of experiment. N.S; not significant. * p <0.05 indicating a significant difference between groups.

**Supplementary Figure 2. CCl4-induced liver fibrosis at the start of lenvatinib treatment.**

(Left panel) Representative microphotographs of Sirius-Red and α-SMA at the start of lenvatinib treatment (Day 14) in Corn-oil (C/O)- or CCl4-mediated rats. Scale bar; 100 μm. (Right panel) Semi-quantitation of Sirius-Red-stained fibrotic area and α-SMA immuno-positive area in high-power field (HPF) by ImageJ software. ** p <0.01 indicating a significant difference between groups. Data are mean ± SD (n=10). Histochemical quantitative analyses included five fields per section. Quantitative values are relatively indicated as fold changes to the values of C/O.

**Supplementary Table 1. List of primers used in q-PCR**

| gene | Sense (5’-3’) | Antisense (5’-3’) |
| --- | --- | --- |
| **Rat** | | |
| *Col1a1* | TGCTGCCTTTTCTGTTCCTT | AAGGTGCTGGGTAGGGAAGT |
| *Ctgf* | AAATAAACTGCCTCCCAAACCA | GAAATGGCTTGCTCAGGGTAAC |
| *Tgfb1* | CGGCAGCTGTACATTGACTT | AGCGCACGATCATGTTGGAC |
| *Cd31* | CCAGAAAGACAAGGCGATCG | CGGCTGGAGGAGAGTTCTAG |
| *Vegfa* | TTCCTGTAGACACACCCACC | TCCTCCCAACTCAAGTCCAC |
| *Vegfr1* | TGCAGGAAACCATAGCAGGA | GTATAGTCCCCTGCGTCCTC |
| *Vegfr2* | CAACGTGGGGCTTGATTTCA | CGCTGTGCAGGTGTATTCTC |
| *Pdgfb* | ATCGAGCCAAGACACCTCAA | ATCACTCCAAGGACCCCATG |
| *Pdgfrb* | AACTCTTCTACCGCTGTGCT | ACAGCAACAATTGGCCTCTG |
| *Fgf2* | CATTCCTGGCCTCTGTCTCC | GCAACTTTCTCCCTTCCTGC |
| *Fgfr2* | CCAGCACCTGTGAGAGAGAA | TTGGAGTTCATGGACGAGCT |
| *Gapdh* | AGACAGCCGCATCTTCTTGT | CTTGCCGTGGGTAGAGTCAT |
| **Human** | | |
| *COL1A1* | CCAAATCTGTCTCCCCAGAA | TCAAAAACGAAGGGGAGATG |
| *ACTA2* | TTCAATGTCCCAGCCATGTA | GAAGGAATAGCCACGCTCAG |
| *TGFB1* | GGGACTATCCACCTGCAAGA | CCTCCTTGGCGTAGTAGTCG |
| *CCND1* | CCGTCCATGCGGAAGATC | ATGGCCAGCGGGAAGAC |
| *CDK4* | CCCACACAAGCGAATCT\CTG | ACCCTCCATAGCCTCAGAGA |
| *CDK6* | AGGCATTTTGGGAACTGTTG | TCCCATCCACTTCAAAGGAG |
| *CDC25A* | GAGATCGCCTGGGTAATGAA | TGCGGAATTCTTCAGGTCT |
| *GAPDH* | CCAAGGAGTAAGACCCCTGG | TGGTTGAGCACAGGGTACTT |
